# Piezoresistive effects in controllable defective HFTCVD graphene-based flexible pressure sensor

**DOI:** 10.1038/srep14751

**Published:** 2015-10-01

**Authors:** Muhammad Aniq Shazni Mohammad Haniff, Syed Muhammad Hafiz, Khairul Anuar Abd Wahid, Zulkarnain Endut, Hing Wah Lee, Daniel C. S. Bien, Ishak Abdul Azid, Mohd. Zulkifly Abdullah, Nay Ming Huang, Saadah Abdul Rahman

**Affiliations:** 1 Nanoelectronics Lab, MIMOS Berhad, Technology Park Malaysia, Kuala Lumpur 57000, Malaysia; 2 School of Mechanical Engineering, USM Engineering Campus, Universiti Sains Malaysia, Nibong Tebal, Pulau Pinang 14300, Malaysia; 3 Low Dimensional Materials Research Centre, Physics Department, Faculty of Science, University of Malaya, Kuala Lumpur 50603, Malaysia; 4 Mechanical Section, Universiti Kuala Lumpur Malaysian Spanish Institute, Kulim Hi-TechPark, Kedah 09000, Malaysia

## Abstract

In this work, the piezoresistive effects of defective graphene used on a flexible pressure sensor are demonstrated. The graphene used was deposited at substrate temperatures of 750, 850 and 1000 °C using the hot-filament thermal chemical vapor deposition method in which the resultant graphene had different defect densities. Incorporation of the graphene as the sensing materials in sensor device showed that a linear variation in the resistance change with the applied gas pressure was obtained in the range of 0 to 50 kPa. The deposition temperature of the graphene deposited on copper foil using this technique was shown to be capable of tuning the sensitivity of the flexible graphene-based pressure sensor. We found that the sensor performance is strongly dominated by the defect density in the graphene, where graphene with the highest defect density deposited at 750 °C exhibited an almost four-fold sensitivity as compared to that deposited at 1000 °C. This effect is believed to have been contributed by the scattering of charge carriers in the graphene networks through various forms such as from the defects in the graphene lattice itself, tunneling between graphene islands, and tunneling between defect-like structures.

Graphene, a two-dimensional (2D) carbon material consisting of hexagonally packed carbon atoms bonded by sp^2^ bonds is the most robust material known. Since the discovery of graphene by Novoselov and Geim in 2004, immense efforts have been focused on potential applications of graphene in optoelectronics, flexible electronics, transparent conducting electrodes, and sensors^1^. These can be made possible through the exceptional properties of graphene, such as its high transmittance (of over 90%), high electron mobility at room temperature (~250000 cm^2^/Vs), very large surface area of 2630 m^2^/g and high elastic stiffness of 340 N/m with a stretchability of up to 20%^2^,^3^. Graphene can be synthesized using a chemical vapor deposition (CVD) technique to produce a large area of uniformed polycrystalline graphene on a transition metal substrate such as copper (Cu) or nickel (Ni). Although it is prerequisite that the complete process typically requires the transfer of graphene from the transition metal support to a target substrate, the production of square meters of graphene on various substrates has already been demonstrated^1^. As the understanding of the graphene growth mechanism by the CVD technique, it has been elucidated that the quality, grain size, number of layers, and morphology of the synthesized graphene can be controlled by many factors such as the substrate temperature, deposition time, pressure, type of substrate, and gas composition^2^,^4^.

Graphene has been popularly exhibited as a sensing material for sensor devices due to its excellent mechanical and electrical properties. A miniature fiber tip pressure sensor that uses graphene as a diaphragm was demonstrated in order to enhance the sensitivity. However, only the pressures in a small range (0 to 13 kPa) could be measured due to leakage, which resulted in the non-ideal adhesion of the graphene layer to the silica capillary endface^5^. Meanwhile, a silicon nitride (SiN_x_) suspended membrane pressure sensor with graphene meander patterns, working in a broad pressure regime of 0–70 kPa, was fabricated by *Zhu et al.*^3^. The fabricated pressure sensor is based on rigid silicon substrate. Therefore, they are not flexible, more fragile and rely on high-cost silicon fabrication processes. Flexibility, highly sensitive and low-cost pressure sensors are highly desirable because of potential applications in structural health monitoring and medicine, which require sensors to be placed in intimate contact with non-planar and curved surfaces (for example human skins). This was realized in a recent study^6^, where a flexible graphene nanosheets/polyurethane (PU) sponge pressure sensor was reported to be capable of measuring pressure in the low-pressure range of 0–10 kPa. However, the fabricated sensor suffers from a nonlinear output response.

Recently, piezoresistive sensing mechanism, which translates a mechanical displacement into an electrical signal, has been widely used because of its advantages, which include feasible preparation, low cost, and easy signal collection^3^. This is also useful for monitoring minute structural deformations in flexible supporting layers over time. In addition, the piezoresistive effect in graphene has been widely used in the MEMS smart sensor field such as for strain sensors^7^,^8^. *Pereira et al.*^9^ proposed that the piezoresistive effect in graphene is related to the graphene lattice distortion, which leads to a modified electronic band structure. However, it is well known that pristine graphene has small resistance changes under strain, with a reported gauge factor of 1.9, due to its semi-metallic behavior^10^. Hence, it may not appear to be a favorable sensing element applicable for monitoring very small changes in structural deformations. Recently, advance investigations on graphene film technology have been conducted by scientists to further enhance the sensor sensitivity. It has been demonstrated that the piezoresistive effect of graphene can be tuned by changing the density of graphene flakes, widening the tunneling between graphene islands, or inducing a non-flat graphene ripple structure^10^,^11^. By considering this morphological effect, it is therefore crucial to understand the piezoresistive mechanisms in graphene that can improve the sensitivity of a pressure sensor.

In this context, we chose to explore the role of graphene defects on the pressure sensing performance and incorporate these results to produce highly sensitive flexible pressure sensors. The graphene was synthesized using a hot-filament thermal chemical vapor deposition (HFTCVD) technique by varying the deposition temperature (750, 850 and 1000 °C) to induce defects in the graphene films in a controlled manner. This technique was utilized because of the higher temperature ramping rate, which has great potential for the large-scale, energy efficient, and rapid manufacture of graphene, and also for producing excellent step coverage and uniform films^12^,^13^. The graphene was then transferred using a wet transfer method mediated by poly-methyl methacrylate (PMMA) onto interdigitated electrode (IDE) in order to improve the flexibility of the substrate and the electrical properties of the graphene network (Supplementary Information, S1). Prior to device characterization, the morphology and electrical properties of the as-transferred graphene as a function of the growth temperature were examined.

## Experimental Procedures

### Hot-filament thermal chemical vapor deposition (HFTCVD)

Graphene was fabricated on Cu foils (25 μm thick, 99.99% purity from Alfa Aesar) in a custom-made HFTCVD system^14^. An alumina tube with one end sealed was wound with a tungsten (W) filament (99.95% purity, 0.5 mm diameter from Kurt J. Lesker) with the substrate rolled and fitted inside the alumina tube. A thermocouple was placed under the Cu foils to monitor the substrate temperature throughout the fabrication process. The filament was then hung across two copper rods, which were connected to an external power supply. The base pressure of the system was 5.0 × 10^−5 ^mbar. Prior to the fabrication process, the W hot-filament was set at ~1750 °C by using an external optical pyrometer and substrate temperature at 1000 °C. A cleaning treatment was performed on the Cu foil in H_2_ at a flow rate of 50 sccm at 3.3 × 10^−1 ^mbar for 20 min to remove the native oxide and increase the Cu grain boundaries. Then, the filament temperature was adjusted to the required temperature of ~1550, ~1650 or ~1750 °C to set the substrate temperature at 750, 850 or 1000 °C, respectively, for the three sets of films studied in this work. The growth of the graphene films was carried out in a CH_4_/H_2_ (50 sccm for CH_4_ and 10 sccm for H_2_) gas mixture for 30 min at a pressure of 3.7 × 10^−1^ mbar. Finally, the samples were slowly cooled to ~200 °C (cooling rate: ~2 °C) by reducing the external power supply and were convectively cooled to room temperature in N_2_ gas at 100 sccm.

### Fabrication method of Interdigitated Electrode

**Figure 1:**
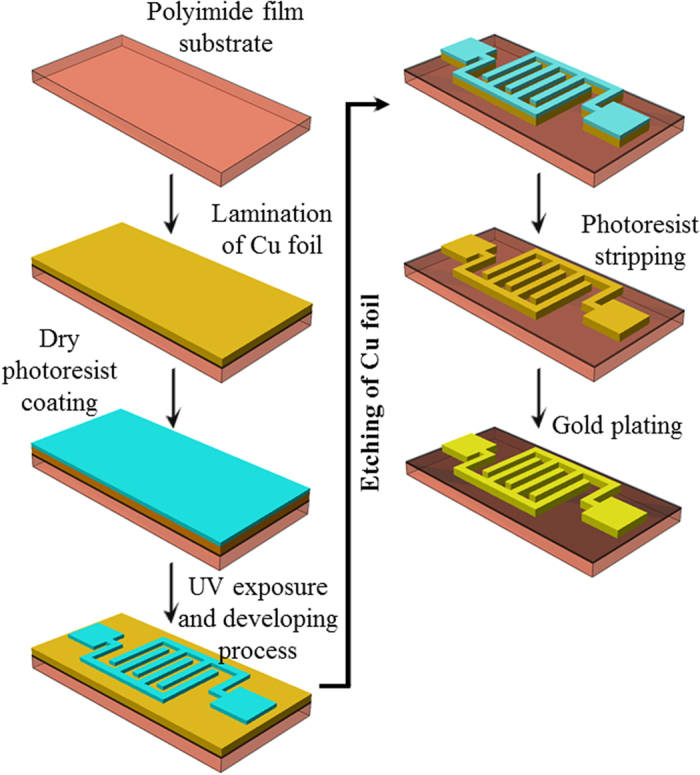
Fabrication process flow of the IDE array on the polyimide substrate film by UV photolithography method.

The fabrication method of interdigitated electrode (IDE) array on polyimide film substrate is presented in Fig. 1. The IDE array, made of the Cu foil, was patterned on a polyimide film substrate using a standard subtractive process based on UV photolithography, in which the unwanted Cu was removed to leave the desired Cu pattern. The Cu foil was adhered to the polyimide film by using an adhesive applied with heat and pressure in a laminating press. The adhesive epoxy resin was coated onto polyimide film (DuPont Kapton® 200HN), and then was laminated to 35 μm-thick Cu foil at a temperature of 160 °C and pressure of 10 MPa for 1 hour in a vacuum condition. The thickness of the composite film was determined to be about ~105 μm. By using the standard UV photolithography method, the Cu foil was first coated with a dry photoresist film (DuPont Riston® GM120) by roll-to-roll lamination at a temperature of 55 °C and a pressure of 0.5 MPa. The photoresist was then exposed in UV light through a mask for 120 seconds and developed in 0.85 wt% sodium carbonate (Na_2_CO_3_). The undeveloped photoresist was etched away. Then, the unwanted, exposed portions of the Cu were removed in a 1.0 mol ferric chloride etching solution. After the exposed Cu was completely etched away, the remaining photoresist was dissolved slowly in a 3 wt% NaOH stripping solution for 120 seconds to create an IDE array with 500-μm tracks and 200-μm gap widths and two terminal electrodes. The Cu patterns were then finalized with Au electroplating with 0.3 ± 0.02 μm to provide a substantial stable nature towards oxidation.

### Morphology characterization

The surface morphology of fabricated graphene-based flexible pressure sensor was characterized by optical microscopy (Olympus MX80), field emission scanning electron microscopy (JEOL JSM-7500F) at a 2-kV accelerating voltage, and atomic force microscopy in semi-contact mode (NTEGRA Spectra, NT-MDT). In addition, a Raman spectroscopy system (Renishaw InVia micro Raman) was utilized to obtain the Raman spectra of the films to determine the thickness and crystalline quality of the graphene layers. The Raman spectra were excited by a 514-nm laser (~2 μm spot size) at a resolution of 2 cm^−1^ in the range of 1000–3000 cm^−1^ and were calibrated using the 520.5 cm^−1^ line of a silicon wafer.

### Flexible pressure sensor with integrated interdigitated electrode (IDE)

**Figure 2:**
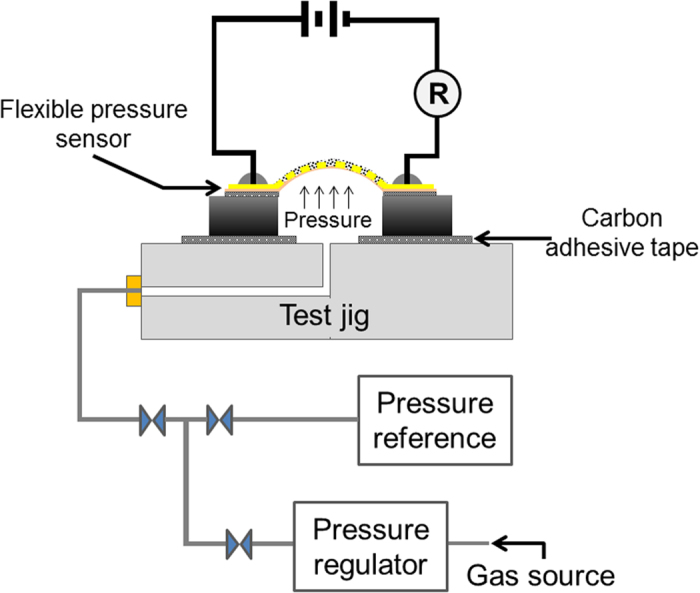
Schematic diagram of a pressure sensor set up.

In order to develop a flexible pressure sensor, the as-transferred graphene sheet was incorporated onto an effective sensing area of 5 × 5 mm^2^ with patterned IDE/polyimide film substrate. To demonstrate the pressure sensing performance, the fabricated sensor was initially attached to the substrate with a square cavity area of 5 × 5 mm^2^ using epoxy. It was then sealed and clamped completely on a test jig by epoxy bonding and carbon tape to prevent gas leakage (see Fig. 2). A differential applied pressure of up to 50 kPa from an N_2_ gas supply system to the cavity was controlled and monitored using an ultralow pressure regulator and a reference pressure sensor (Vernier, gas pressure sensor). The length of pipeline from the valve to the fabricated pressure sensor and the commercial pressure sensor was fixed at 20 cm. In this case, the applied pressure on the fabricated pressure sensor was assumed to be equal to the measurement of the reference pressure sensor. Both the diaphragm and graphene experienced deformation under the applied pressure (Supplementary Information, S2), and the resistance changes were simultaneously measured using a source-meter (EA4980A Agilent, LCR meter) at ambient conditions.

## Results and Discussion

### Morphology characterization

**Figure 3:**
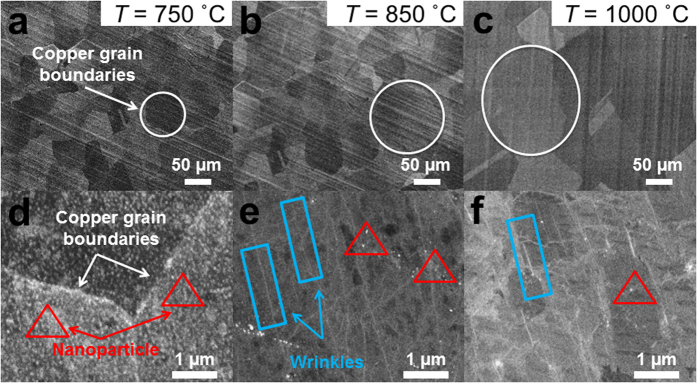
FESEM images of graphene prepared by HFTCVD on Cu foil substrate at various growth temperatures: (a) 750, (b) 850 and (c) 1000 °C. Higher magnification of FESEM images was shown in (**d**–**f**) for 750, 850 and 1000 °C growth temperatures, respectively. The circle, triangle and rectangular shapes indicate Cu grain domain sizes, nanoparticles and wrinkles.

Figure 3(a–c) shows the FESEM images of the as-grown graphene on Cu foil substrates at different growth temperatures. It was observed that the Cu grain boundaries increased from ~50 μm to more than ~200 μm as the substrate temperature was increased from 750 to 1000 °C. This has been documented frequently for the growth of graphene on Cu foils; an annealing process at ~1000 °C will be done prior to the deposition process to increase the copper grain boundary and decrease surface roughness. This effect was more prominent when the temperature was close to the melting point of copper (~1083 °C), where the growth of larger graphene grains was preferable. For many of the properties of current interest, such as electronic mobility and thermal conductivity, large copper grain boundaries are desirable to produce a lower graphene nucleation density, and vice versa. A decrease in graphene nucleation density would result in larger graphene grain sizes^4^,^15^. This is attributed to the higher adsorption of hydrocarbon onto the Cu surface, which consequently led to a denser nucleation at a relatively low temperature. In addition, the structural defect was observed to be in the form of wrinkles and nanoparticles, which were dominant for graphene grown at 750 °C (see Fig. 3(d–f)). The nanoparticle has been identified in our previous work as Cu_2_O, which acts as nucleation site for graphene^14^.

**Figure 4:**
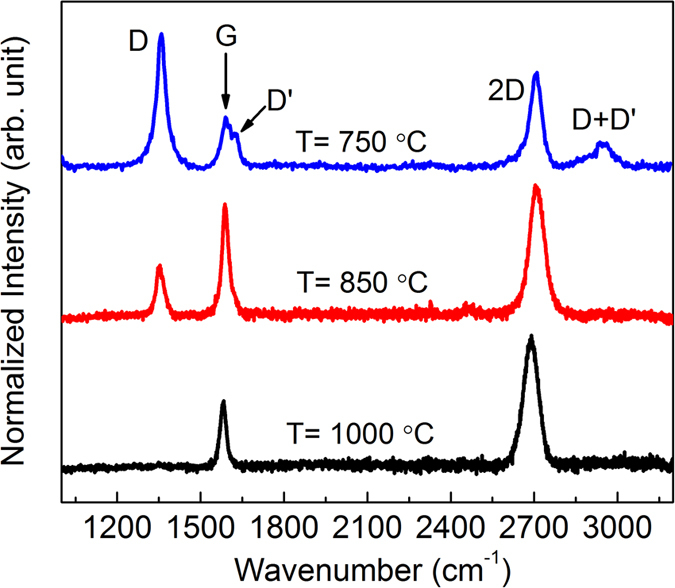
Raman spectra of graphene grown on Cu foil substrate as function of substrate temperature.

Figure 4 shows the Raman spectra of the graphene films on the Cu foil substrate. All the spectra show the three major Raman peaks for graphene: the D, G, D’, 2D and D+D’ peaks located at ~1359, ~1590, ~1620, ~2700 and ~2949 cm^−1^, respectively (Supplementary Information, S3). From the results, it can be seen that the evolution of the D peak shows the strong temperature dependence of the substrate temperature (I_D_/I_G_ ratio of 750, 850 and 1000 °C is 1.24, 0.77 and ~0.01, respectively), suggesting a major change in the defect concentration in the graphene. The intensity of the I_D_/I_G_ ratio has often been used to identify the defect density inside graphene, given that a higher ratio indicates more defects and vice versa. This is probably due to the evolutionary kinetics of the graphene formation on the copper substrate as a result of the effects of the adsorption, dissociation, dehydrogenation and copper sublimation^15^. The monolayer graphene growth is determined by using the I_2D_/I_G_ ratio value, where an I_2D_/I_G_ larger than 1.5 indicates monolayer graphene growth on copper substrate, and good correlations have been reported in the literature^14^,^16^.

**Figure 5:**
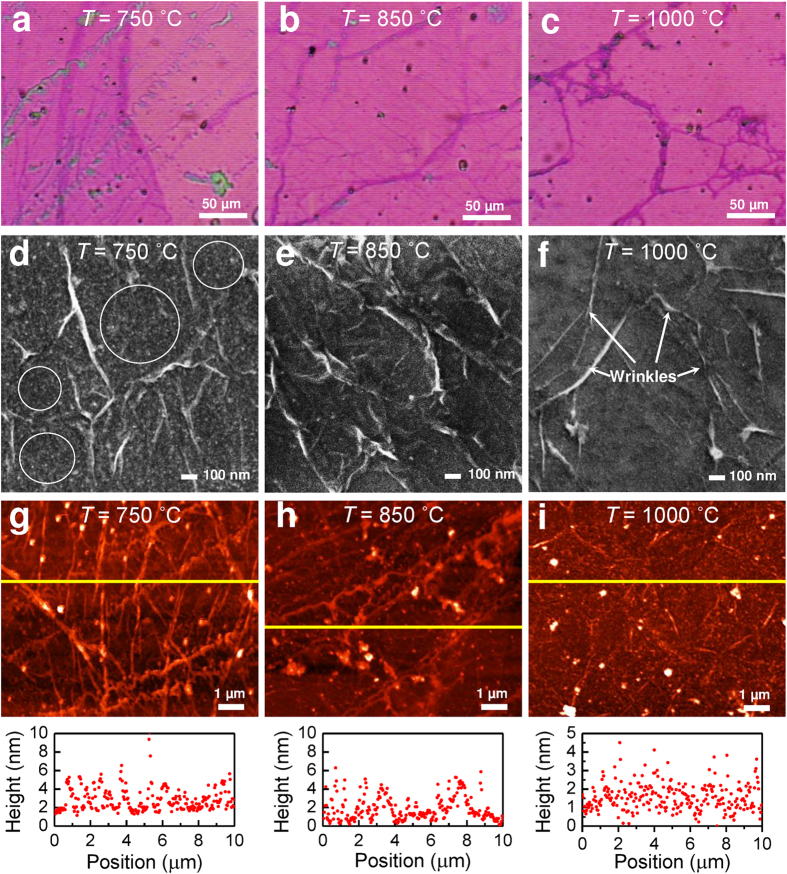
Optical, FESEM, and AFM images of transferred graphene onto fabricated pressure sensors. (**a**–**c**) Optical microscopy images of resultant transferred graphene at different substrate temperatures. (**d**–**f**) FESEM images at high magnification (50,000×) and (g-i) the corresponding AFM images with *x*-cross section profiles for graphene grown at 750, 850 and 1000 °C, respectively.

Further, these three morphologies of graphene films were selected to develop flexible pressure sensors. The morphological characteristics of the resultant transferred graphene on the sensor platform are shown in Fig. 5. The optical image of graphene in Fig. 5(a) shows a color contrast between the overlapping of left and right sides of the graphene sheets, which represents the difference in the numbers of graphene layers deposited at these sites. The darker region of the color contrast is due to the overlapping graphene layers. This is similar to the slightly folded graphene, which appears as wrinkles in the image. On the other hand, the transferred graphene grown at the 850 °C and 1000 °C substrate temperatures (see Fig. 5(b,c)) do not show this color contrast scheme, indicating less probability of deposited graphene layers overlapping, due to a lower nucleation density. It was also observed that the presence of defect-like structures such as wrinkles and nanoparticles was more prominent in graphene grown at 750 °C compared to that grown at higher growth temperatures (see Fig. 5(d–f)). This correlates well with the Raman analysis shown in Fig. 4. At a lower growth temperature, these nanoparticles could easily be induced at nucleation sites as defective structures during the initial stage of graphene growth due to the lower desorption rate of carbon atoms onto the Cu surface. However, these could also be from the residue of copper vapor that was simultaneously deposited on the graphene layer as a nanoparticle. In addition, the RMS (root mean square) surface roughness of the resultant transferred graphene was shown to increase by ~50% when the growth temperature decreased from 1000 to 750 °C due to the presence of a larger defect density (see Table 1). In this instance, the results showed that the morphological evolution of graphene with different defect densities could be tuned by changing the graphene growth temperature.

### Electromechanical characterization

The transport properties of the transferred graphene on the SiO_2_/Si (100) substrate with patterned Pt electrodes (thickness, *d *≈ 100 nm) were studied using the Van der Pauw method. The graphene channel’s width and length were fixed equally at 1000 μm. The contact resistance of the Pt electrodes was found to be ~3.0 Ω. The transferred graphene for a growth temperature of 750 °C exhibited a higher sheet resistance of about 1222 ± 18.3 Ω/sq. (ohms per square), but this decreased to 166 ± 6.4 Ω/sq. as the growth temperature increased to 1000 °C, as listed in Table 1. It was found that the measured sheet resistance of the transferred samples could be improved by increasing the growth temperature of the graphene. This can be explained by the morphological evolution of the graphene film, which considered the graphene grain size, the effects of topological defects such as dislocations, grain boundaries, wrinkles, and cracks^17^,^18^, and also the possibility of residual chemical impurities^19^ from the as-transferred graphene or growth processes, which could effectively scatter the charge carriers through the graphene percolating networks. Our transferred graphene could achieve a lower sheet resistance of ~166 Ω/sq., but the value obtained was slightly higher compared to that of the theoretical value of pristine graphene (30 Ω/sq.) due to the scattering from defects^20^. However, compared to the high defective-CVD growth of graphene by *Gao et al.*^21^, the transferred graphene grown at a substrate temperature of 750 °C still had better sheet resistance (~1.2 kΩ/sq.) than that of the transferred graphene grown at 1000 °C (~1.7 kΩ/sq.).

**Figure 6:**
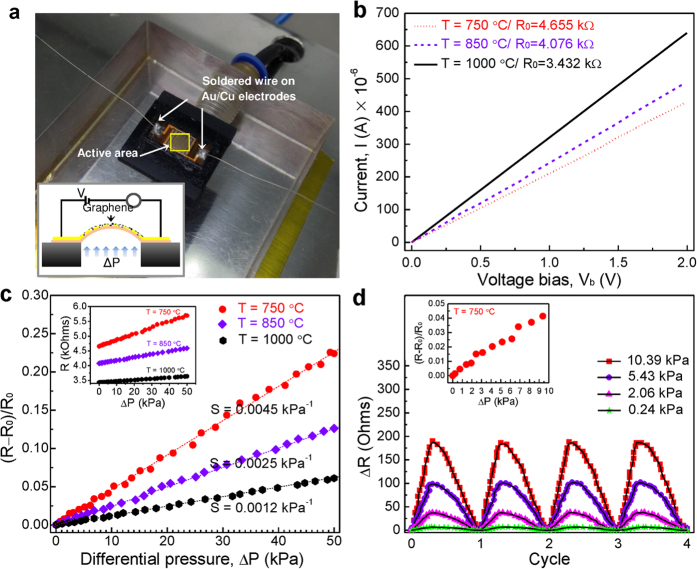
(**a**) Photograph of fabricated graphene-based flexible pressure sensor fixed in test jig at ambient conditions. The inset shows a scheme of the graphene-based pressure sensor characterization setup. (**b**) A plot of the *I*–*V* curve characteristics. (**c**) Relative change in resistance as a function of applied differential pressure. The inset shows that the resistance increases as the differential pressure increases. (**d**) Multi-cycle operation of repeated loading and unloading at different pressures. The inset shows a plot of the relative change in resistance in the low-pressure regime (<10.4 kPa) for a flexible pressure sensor with graphene grown at 750 °C.

Prior to testing the performance of the sensor devices to applied pressure (see Fig. 6(a)), their initial resistances were measured at ambient conditions and had values of 4.655, 4.076 and 3.432 kΩ for the graphene growth temperatures of 750, 850 and 1000 °C, respectively. In order to verify the consistency of the initial resistance value, further characterization of the *I*-*V* curve was performed, where the current increased linearly as the bias voltage increased from 0 to 2 V, as shown in Fig. 6(b). It should be noted that each of the transferred graphene onto devices displayed stable electrical properties in the ohmic characteristics. In order to demonstrate the effects of graphene defects on the pressure sensitivity, the relative change in the resistance of the graphene-incorporated pressure sensor with the applied differential pressure was investigated with different degree of defect density. Figure 6(c) shows the results for the relative change in resistance as a function of the applied differential pressure. The sensitivity of the pressure sensor can be defined as *S *= (Δ*R*/*R*_*0*_)/Δ*P*) and the unit is kPa^−1^, in which Δ*R* is the change in resistance (Ω), *R*_*0*_ is the initial resistance (kΩ), and Δ*P* is the pressure difference (kPa). The sensitivities for graphene grown at 750, 850 and 1000 °C based on the slope of the fitted lines can be calculated as 0.0045, 0.0025 and 0.0012 kPa^−1^, respectively. These experimental results demonstrate that this piezoresistive effect in the graphene networks could be improved by lowering the graphene growth temperature.

In the present work, it has been successfully demonstrated that a higher sensitivity of sensor device can be achieved by utilizing highly-dense graphene defects as compared to previous works demonstrated for flexible pressure sensor such as the rGO-polyurethane sponge (0.001 kPa^−1^)^6^, carbon black-silicone rubber nanocomposite (0.001 kPa^−1^)^22^, and MWCNT-polyimide nanocomposite (~0.00025 kPa^−1^)^23^. Multiple tests by loading and unloading pressure at different values were carried out to evaluate the robustness of the piezoresistive effect. It could be observed that the flexible pressure sensor was able to detect pressures as low as 0.24 kPa with a good resistance change of ~23 mΩPa^−1^, which is comparable to the sensitivity of a discrete pressure sensor. The results of repeated cycling are shown in Fig. 6(d).

**Figure 7:**
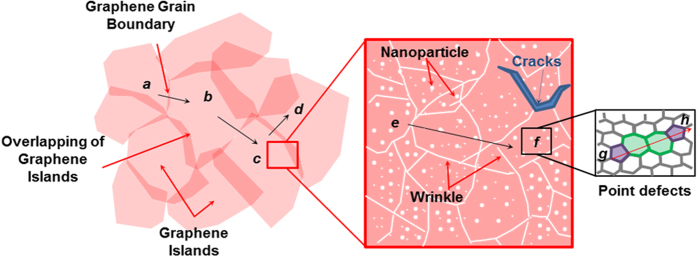
Schematic representations of the proposed resistance mechanism in defective-graphene network.

For pristine graphene grown at a higher growth temperature, the piezoresistive effect under deformation is limited to the structure deformation-dependence of the graphene lattice distortion, which only leads to a modified electronic band structure, so the overall variation of resistance is smaller^24^. Compared with pristine graphene, the transport of charge carriers in the present work was different and the sensing mechanism was not limited to the change in the carbon-carbon bonds length, but also depended on the defective structure of the graphene networks. Here, four mechanisms are emphasized to explain the resistance of defective graphene (see Fig. 7): contact resistance by direct contact between adjacent graphene grains (from *a* to *b*) or their overlap area (from *c* to *d*); resistance by tunneling or electron hopping effect between neighboring graphene islands (from *b* to *c*); resistance by tunneling effect through defects-like line disruptions (from *e* to *f*); and resistance by point defects-like vacancies or substitutional impurities (from *g* to *h*). In view of that, measurable changes in the resistance can be predicted by exploiting the aforementioned mechanisms which could result from even small modifications to the lattice distortion in the graphene itself, contact area or tunneling distance between neighboring graphene islands with structural defects.

**Figure 8:**
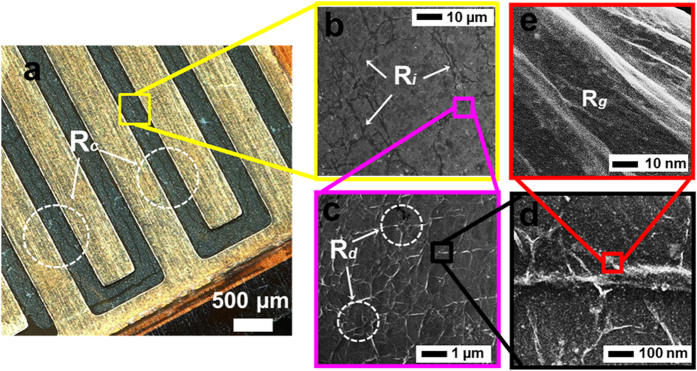
(**a**) Optical image of the transferred graphene onto a flexible IDE/polyimide film substrate. (**b**–**d**) FESEM images of as-transferred graphene on the sensor device at substrate temperature of 750 °C with different possibilities for resistance changes.

Figure 8(a) shows an image of the transferred graphene that was fully enclosed in the IDE structure. For the as-fabricated sensor device with highly defective-graphene grown at 750 °C, it is predicted that the higher initial resistance is mainly attributed to the scattering of charge carriers due to smaller graphene islands with topological defects. In this case, the contribution of resistance changes in these graphene networks can be related to the tunneling between graphene grains (R_*i*_), tunneling between the defect-like line disruptions structure (R_*d*_), defects in the graphene lattice itself (R_*g*_) and the contact between the graphene and IDE array (R_c_). Hence, any changes to these parameter as a function of applied pressure would induce strained in graphene lattice structure and scattering of charge carriers from the increased in tunneling distance, thus resulting in a significant resistance change (see Fig. 8(a–d)), which can be defined as ΔR = ΔR_c_ + ΔR_*i*_ + ΔR_*d*_ + ΔR_*g*_. It should be noted that there is also the probability that changes of resistance might come from the graphene that is directly touching the IDE, causing a contact resistance effect when pressure is applied to the flexible substrate. However, if the contact resistance effect is significant compared to the difference of defect density in graphene, the response of the pressure sensor might not be linearly dependent on the applied pressure (Fig. 6(c)) and no multi-cycle of repeated loading and unloading at different pressures (Fig. 6(d)) can be achieved^6^. Thus, the contact resistance effect as a function of applied pressure can be neglected in the discussion.

**Figure 9:**
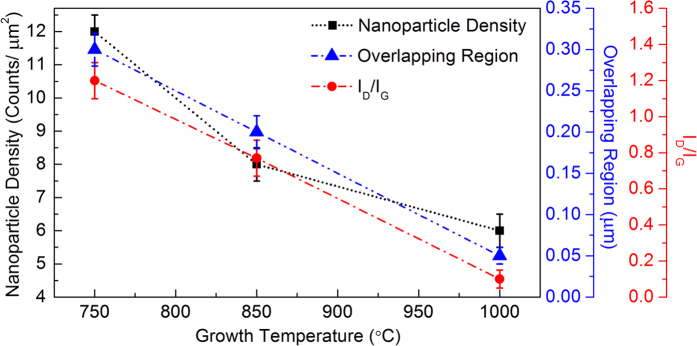
Nanoparticle density (Counts/μm^2^) in 10 × 10 μm area, overlapping region (μm) between adjacent graphene island and I_D_/I_G_ measured from AFM, FESEM and Raman spectroscopy, respectively.

The concentration of the defects within the graphene layer which relates to the nanoparticle density, overlapping region and I_D_/I_G_ ratio was presented in Fig. 9. The nanoparticle density was measured from the AFM images (refer to Fig. 5(g–i)) in 10 × 10 μm^2^ with the count of nanoparticle significantly decrease from 1160, 768 and 547 nanoparticles at 750, 850 and 1000 °C, respectively. In addition, the overlapping region of adjacent graphene islands was observed from the FESEM images (Supplementary Information, S4) to be at 0.30, 0.20 and ~0.05 μm at 750, 850 and 1000 °C, respectively. From the graph, the concentration of the defects within the graphene layer is inversely proportional to the growth temperature. It has been established that based on the tunneling mechanism, a larger tunneling distance under applied strain or pressure would enhance the change in the resistance of the graphene material. In this work, it has been demonstrated that the same characteristic is also valid for the fabricated graphene-based pressure sensor, but with further scattering effects on localized defects supporting line disruptions and dislocations. At a growth temperature of 750 °C, the deformation of the high defect density had a more prominent influence on the resistance changes than that of perfect graphene due to the quantum confinement effects in the presence of point defects and line disruption defects. The strong dependence of the resistance changes in the high defect density of the graphene networks on the structural deformation could be attributed to the creation of charge carrier scattering. Under the application of pressure, the tunneling distortion among overlapped and adjacent graphene islands and defect-like structures became more significant, which in turn increased the density of the tunneling per graphene area. This led to a higher probability of electron scattering in the percolating networks, which further reduced the density of the conducting paths, thereby effectively increasing the rate of the resistance change. On the other hand, different percolation schemes through specific type of defects (i.e. nanoparticle density, overlapping region or I_D_/I_G_ ratio) may have a significant impact to the piezoresistive effect of graphene-based flexible pressure sensors which can be further study, but these cases exceed the scope of this work.

## Conclusion

In this work, a fabricated flexible pressure sensor incorporating graphene synthesized at various substrate temperatures of 750, 850 and 1000 °C through the hot-filament thermal chemical vapor deposition (HFTCVD) technique has been successfully demonstrated. The experimental results show that the graphene grown at a lower temperature has a higher structural-defect density as compared to those grown at a higher substrate temperature. A linear piezoresistive effect has been observed for the applied pressure within the range of 0 to 50 kPa. The sensitivity of the fabricated pressure sensor could easily be modified by varying the state of the substrate temperature. In this work, the highest sensitivity of 0.0045 kPa^−1^ has been attained for the sensor using graphene grown at the lowest substrate temperature of 750 °C. However, the sensitivity could be further enhanced by patterning smaller IDE channel or lowering the thickness of the diaphragm. This investigation provided a better understanding of the use of graphene in highly sensitive piezoresistive-based flexible pressure sensors.

## Additional Information

**How to cite this article**: Mohammad Haniff, M. A. S. *et al.* Piezoresistive effects in controllable defective HFTCVD graphene-based flexible pressure sensor. *Sci. Rep.* **5**, 14751; doi: 10.1038/srep14751 (2015).

## Supplementary Material

Supplementary Information

## Figures and Tables

**Table 1:** Summary of Raman spectra on Cu foil, AFM measurements, and electrical properties of transferred graphene on SiO_2_/Si (100) substrates at different growth temperatures.

**Substrate temperature, *T* (°C)**	**Intensity ratio, (I_D_/I_G_)**	**RMS-roughness (nm)**	**Sheet resistance (Ω/sq.)**
750°C	1.24	7.85	1222 ± 18.3
850°C	0.77	6.72	761 ± 9.9
1000°C	~0.01	3.95	166 ± 6.4

## References

[b1] Novoselov K. S. *et al.* A roadmap for graphene. Nature 490, 192–200 (2012). 23060189 10.1038/nature11458

[b2] Mattevi C., Kim H. & Chhowalla M. A review of chemical vapour deposition of graphene on copper. J. Mater. Chem. 21, 3324–3334 (2011).

[b3] Zhu S.-E., Krishna Ghatkesar M., Zhang C. & Janssen G. C. A. M. Graphene based piezoresistive pressure sensor. Appl. Phys. Lett. 102, 161904 (2013).

[b4] Seah C.-M., Chai S.-P. & Mohamed A. R. Mechanisms of graphene growth by chemical vapour deposition on transition metals. Carbon 70, 1–21 (2014).

[b5] Ma J., Jin W., Ho H. L. & Dai J. Y. High-sensitivity fiber-tip pressure sensor with graphene diaphragm. Opt. Lett. 37, 2493–2495 (2012). 22743432 10.1364/OL.37.002493

[b6] Yao H.-B. *et al.* A Flexible and Highly Pressure-Sensitive Graphene–Polyurethane Sponge Based on Fractured Microstructure Design. Adv. Mater. 25, 6692–6698 (2013). 24027108 10.1002/adma.201303041

[b7] Bae S.-H. *et al.* Graphene-based transparent strain sensor. Carbon 51, 236–242 (2013).

[b8] Tian H. *et al.* Scalable fabrication of high-performance and flexible graphene strain sensors. Nanoscale 6, 699–705 (2014). 24281713 10.1039/c3nr04521h

[b9] Pereira V. M., Castro Neto A. H., Liang H. Y. & Mahadevan L. Geometry, Mechanics, and Electronics of Singular Structures and Wrinkles in Graphene. Phys. Rev. Lett. 105, 156603 (2010). 21230923 10.1103/PhysRevLett.105.156603

[b10] Huang M., Pascal T. A., Kim H., Goddard W. A. & Greer J. R. Electronic−Mechanical Coupling in Graphene from *in situ* Nanoindentation Experiments and Multiscale Atomistic Simulations. Nano Lett. 11, 1241–1246 (2011). 21309539 10.1021/nl104227t

[b11] Wang C. Y., Mylvaganam K. & Zhang L. C. Wrinkling of monolayer graphene: A study by molecular dynamics and continuum plate theory. Phys. Rev. B: Condens. Matter 80, 155445 (2009).

[b12] Piner R. *et al.* Graphene Synthesis via Magnetic Inductive Heating of Copper Substrates. ACS Nano 7, 7495–7499 (2013). 23930903 10.1021/nn4031564

[b13] Hawaldar R. *et al.* Large-area high-throughput synthesis of monolayer graphene sheet by Hot Filament Thermal Chemical Vapor Deposition. Sci. Rep. 2, 682 (2012). 23002423 10.1038/srep00682 PMC3448070

[b14] Syed Muhammad H., Chong S. K., Huang N. M. & Abdul Rahman S. Fabrication of high-quality graphene by hot-filament thermal chemical vapor deposition. Carbon 86, 1–11 (2015).

[b15] Celebi K. *et al.* Evolutionary Kinetics of Graphene Formation on Copper. Nano Lett. 13, 967–974 (2013). 23339597 10.1021/nl303934v

[b16] Lu A.-Y. *et al.* Decoupling of CVD graphene by controlled oxidation of recrystallized Cu. R. Soc. Chem. Adv. 2, 3008–3013 (2012).

[b17] Duong D. L. *et al.* Probing graphene grain boundaries with optical microscopy. Nature 490, 235–239 (2012). 23034653 10.1038/nature11562

[b18] Tsen A. W. *et al.* Tailoring Electrical Transport Across Grain Boundaries in Polycrystalline Graphene. Science 336, 1143–1146 (2012). 22654054 10.1126/science.1218948

[b19] Suk J. W. *et al.* Enhancement of the Electrical Properties of Graphene Grown by Chemical Vapor Deposition via Controlling the Effects of Polymer Residue. Nano Lett. 13, 1462–1467 (2013). 23510359 10.1021/nl304420b

[b20] Chen J.-H., Jang C., Xiao S., Ishigami M. & Fuhrer M. S. Intrinsic and extrinsic performance limits of graphene devices on SiO2. Nat. Nano 3, 206–209 (2008). 10.1038/nnano.2008.58 18654504

[b21] Gao L., Ren W., Zhao J., Ma L. P., Chen Z., & Cheng H. M. Efficient growth of high-quality graphene films on Cu foils by ambient pressure chemical vapor deposition. Appl. Phys. Lett. 97, 183109 (2010).

[b22] Luheng W., Tianhuai D. & Peng W. Thin Flexible Pressure Sensor Array Based on Carbon Black/Silicone Rubber Nanocomposite. IEEE Sens. J. 9, 1130–1135 (2009).

[b23] Gau C., Ko H. S. & Chen H. T. Piezoresistive characteristics of MWNT nanocomposites and fabrication as a polymer pressure sensor. Nanotechnology 20, 185503 (2009). 19420615 10.1088/0957-4484/20/18/185503

[b24] Jin C., Lan H., Peng L., Suenaga K. & Iijima S. Deriving Carbon Atomic Chains from Graphene. Phys. Rev. Lett. 102, 205501 (2009). 19519038 10.1103/PhysRevLett.102.205501

