# Supplementary material for: Piezoresistive effects in controllable defective HFTCVD graphene-based flexible pressure sensor
Source: Sci Rep. 2015 Oct 1;5:14751. doi: 10.1038/srep14751 (PMC4589683; doi:10.1038/srep14751)
Supplement: Supplementary Information [file srep14751-s1.doc]

**Supplementary Information**

**Piezoresistive effects in controllable defective HFTCVD graphene-based flexible pressure sensor**

Muhammad Aniq Shazni Mohammad Haniff ^1,2,#,*^, Syed Muhammad Hafiz ^3,#^, Khairul Anuar Abd Wahid ^1^, Zulkarnain Endut ^1^, Hing Wah Lee ^1^, Daniel C. S. Bien ^1^, Ishak Abdul Azid ^4^, Mohd. Zulkifly Abdullah ^2^, Nay Ming Huang ^3,*^, Saadah Abdul Rahman ^3^

^1^Nanoelectronics Lab, MIMOS Berhad, Technology Park Malaysia,

Kuala Lumpur 57000, Malaysia.

^2^School of Mechanical Engineering, USM Engineering Campus,

Universiti Sains Malaysia, Nibong Tebal, Pulau Pinang 14300, Malaysia.

^3^Low Dimensional Materials Research Centre, Physics Department,

Faculty of Science, University of Malaya, Kuala Lumpur 50603, Malaysia.

^4^Mechanical Section, Universiti Kuala Lumpur Malaysian Spanish Institute, Kulim Hi-TechPark, Kedah 09000, Malaysia.

# These authors contributed equally in this work

*Corresponding author 1) [*aniq.haniff@mimos.my*](mailto:aniq.haniff@mimos.my)

2) *syedhafiz27@gmail.com*

3) [*huangnayming@gmail.com*](mailto:huangnayming@gmail.com)

**S1) Graphene transfer method**

The graphene fabricated on the Cu foils was transferred to a flexible polyimide substrate via a wet etching transfer process. This process was assisted by using a thin layer of poly-methyl methacrylate (MicroChem 950 PMMA, 4% in Anisole). Firstly, the PMMA was spin-coated to the as-grown graphene on one of its side to act as a support layer for the graphene sheet. The Cu foil was then removed by immersing the stack of Cu foils in a ferric chloride (FeCl_3_) solution (1.0 mol) for 30 min at room temperature. The etching solution color turned from brownish to dark brownish when the Cu foil was etched. It should be noted that the entire process should be performed in a fume hood as a corrosive vapor is released from the reaction of this process. After the Cu foils were completely etched away, the graphene supported by PMMA was scooped and transferred into deionized water a few times to remove any of the Cu residues. Lastly, the graphene was scooped with a flexible polyimide substrate for further characterization. This transfer process, assisted by the PMMA, was employed because it was capable of transferring a large and uniform area of graphene from a Cu foil to another substrate[^1^](#_ENREF_1). The overall graphene transfer process onto the flexible polyimide substrate with interdigitated electrode (IDE) is schematically shown in **Fig. S1.**


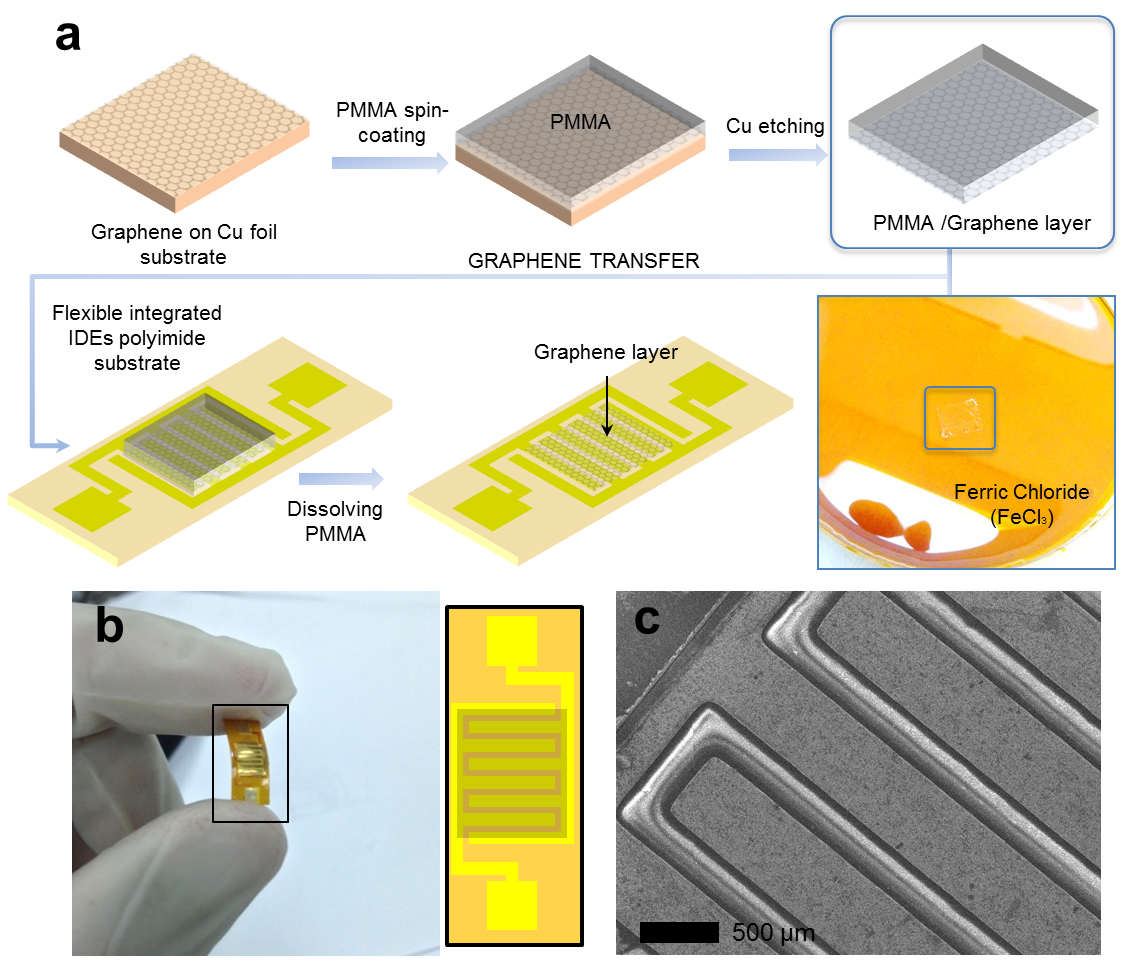


**Figure S1** (a) Schematic representation of graphene transfer process on flexible pressure sensor device. (b) Photograph of flexible pressure sensor with integrated IDE microstructure. The inset shows the design of the IDE. (c) FESEM image of IDE with 500-µm tracks and 200-µm gap widths.

**S2** **Finite element analysis (FEA)**

Finite element analysis (FEA) on the fabricated sensor has been conducted using CoventorWare® to demonstrate that the applied pressure (0 to 10 kPa) on the bottom surface of the fabricated sensor forms a round-shape deformation.


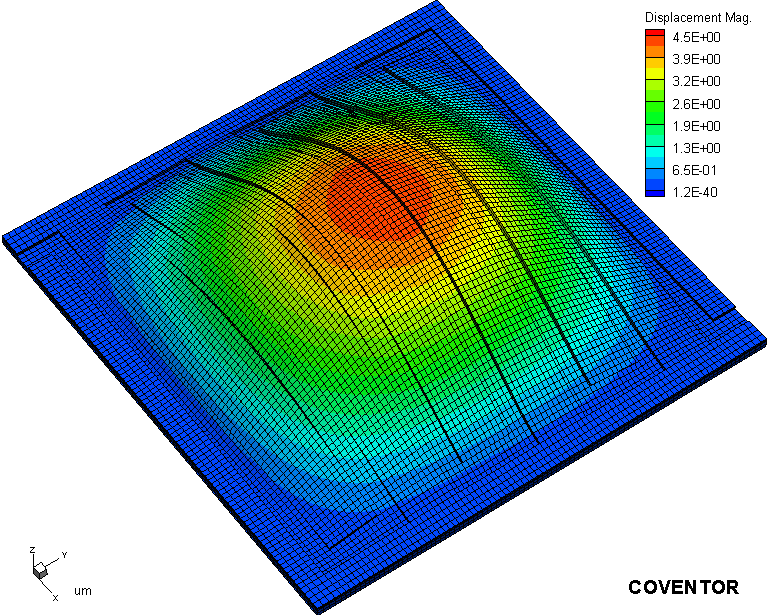


**Figure S2** Finite element analysis (FEA) on the fabricated sensor has been conducted using CoventorWare® to demonstrate that the applied pressure (0 to 10 kPa) (Left) on the bottom surface of the fabricated sensor forms a round-shape deformation (Right).

**S3) Raman characterization analysis**

The D peak is due to the breathing modes of six-atom rings and it comes from TO phonons around the K point of the Brillouin zone center. It is active because of a double resonance (DR) inter-valley process, and the presence of the D band indicates the presence of a large concentration of defects in the graphene. The G peak is associated with the double-degenerate (iTO and LO) phonon mode (E_2g_ symmetry) at the Brillion zone center. The D’ peak occurs via an intra-valley double resonance process in the presence of defects and D+D’ is the combination mode of two phonons with different momentum. The 2D peak originates from a DR (inter-valley process) mechanism, involving two iTO phonons near the K and K’ points in the first Brillion zone of graphene[^2^](#_ENREF_2).

**S4) FESEM images of overlapping region**

Graphene grown at lower growth temperature has higher overlapping region compared to the growth at higher temperature. This is due to the higher nucleation density (nanoparticles) observed at 750 °C growth temperature, that will promotes the graphene island growth closer from each other and have a higher chances of overlapping event. It is evidence from the FESEM images shown in **Fig. S4** that the overlapping region is decreasing from 0.30 µm to ~0.05 µm at 750 and 1000 °C growth temperature, respectively.


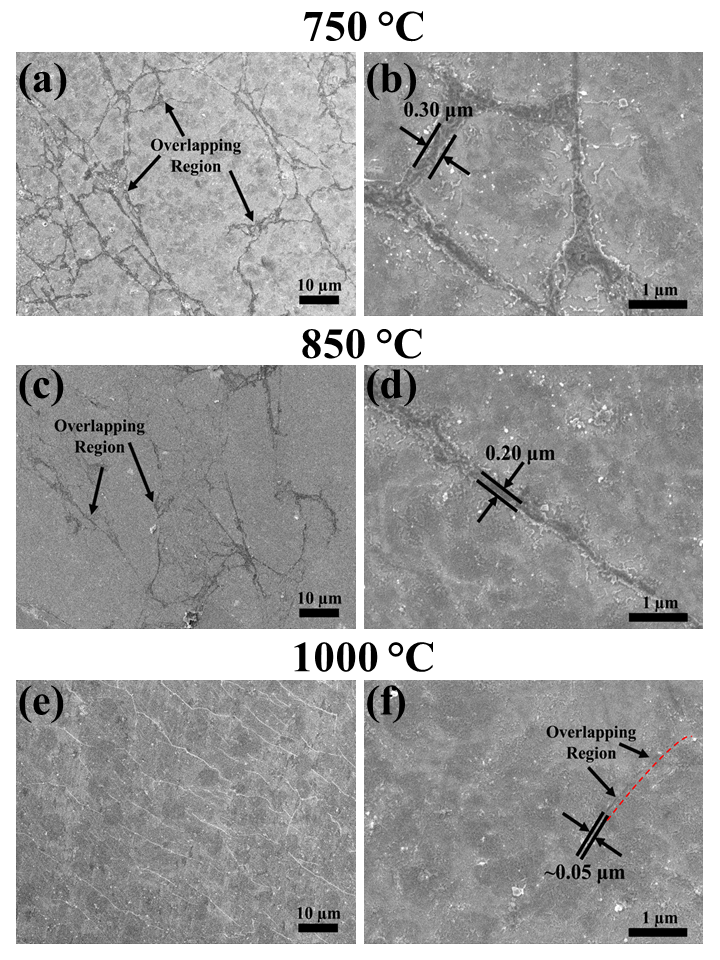


**Figure S4** FESEM images of overlapping region of graphene grown at (a-b) 750 °C (c-d) 850 °C and (e-f) 1000 °C.

**References**

1 Mattevi, C., Kim, H. & Chhowalla, M. A review of chemical vapour deposition of graphene on copper. *J. Mater. Chem.* **21**, 3324-3334 (2011).

2 Malard, L. M., Pimenta, M. A., Dresselhaus, G. & Dresselhaus, M. S. Raman spectroscopy in graphene. *Phys. Rep.* **473**, 51-87 (2009).
